# Kikuchi–Fujimoto disease associated with systemic lupus erythematosus complicated with hemophagocytic lymphohistiocytosis: a case report

**DOI:** 10.1186/s13256-019-2100-1

**Published:** 2019-06-06

**Authors:** Sahathevan Vithoosan, Thilini Karunarathna, Ponnudurai Shanjeeban, Paramarajan Piranavan, Thushara Matthias, Dayal Gamlaksha, Ama Liyadipita, Aruna Kulatunga

**Affiliations:** 1 The National Hospital of Sri Lanka, Colombo 10, Sri Lanka; 2 Saint Vincent Hospital, Worcester, MA 01608 USA; 3 Provincial General Hospital, Ratnapura, Sri Lanka

**Keywords:** Kikuchi–Fujimoto disease, Systemic lupus erythematosus, Hemophagocytic lymphohistiocytosis

## Abstract

**Background:**

Kikuchi–Fujimoto disease, which was originally described in young women, is a benign condition characterized by necrotizing lymphadenitis and fever. Even though the clinical course is usually self-limiting, it can be associated with recurrences and rarely can be associated with systemic lupus erythematosus or can be complicated with hemophagocytic lymphohistiocytosis. We report the case of a 17-year-old Sri Lankan Sinhalese schoolboy who presented with fever and cervical lymphadenopathy diagnosed as Kikuchi–Fujimoto disease and was complicated with hemophagocytic lymphohistiocytosis subsequently. Later he fulfilled the criteria for systemic lupus erythematosus.

**Case presentation:**

A 17-year-old previously healthy Sinhalese schoolboy presented with high-grade fever associated with chills and rigors associated with loss of appetite and loss of weight for more than 40 days. On examination, he had bilateral firm matted tender cervical lymphadenopathy and firm hepatomegaly. An excision biopsy of his right cervical lymph node revealed necrotizing lymphadenitis and immunohistochemistry of a lymph node biopsy favored Kikuchi disease. Initial antinuclear antibody and anti-double-stranded deoxyribonucleic acid tests were negative and his C3 and C4 levels were normal. An infections screening was negative. He was treated with steroids. While in hospital he developed hemophagocytic lymphohistiocytosis and renal impairment. Later his antinuclear antibody titer became positive in 1:160 and fulfilled the diagnostic criteria for systemic lupus erythematosus. He was managed with steroids and immune suppressive drugs and showed remarkable improvement.

**Conclusion:**

Although Kikuchi–Fujimoto disease is uncommon in male patients, it needs to be considered in patients with lymphadenopathy and fever. The disease can be complicated with hemophagocytic lymphohistiocytosis and the patients need continuous monitoring for the possible development of systemic lupus erythematosus later in the course.

## Background

Kikuchi–Fujimoto disease (KFD) is a rare benign disease characterized by fever and cervical lymphadenopathy. It was initially described by Japanese pathologists Kikuchi and Fujimoto in 1972. Its etiology remains unknown even though various environmental and infectious factors have been suspected [1]. Females are affected more often than males, and the disease is more prevalent in Asia. It can be associated with other autoimmune disorders especially systemic lupus erythematosus (SLE) [1]. Three types of associations between KFD and SLE have been reported: KFD before the occurrence of SLE, the simultaneous occurrence of KFD and SLE, and KFD after the occurrence of SLE [2]. Both KFD and SLE are rarely associated with the life-threatening complication, hemophagocytic lymphohistiocytosis (HLH) [3]. Only a limited number of cases are found in the literature showing an association between KFD and SLE in male patients. Here, we report a rare case of KFD in a Sri Lankan boy, which was complicated with HLH and subsequently met the diagnostic criteria for SLE.

## Case presentation

A 17-year-old previously healthy Sinhalese schoolboy presented with high-grade fever associated with chills, rigors, and right-sided cervical lymphadenopathy of 2 weeks’ duration. He also had a loss of appetite following fever onset but there was no significant loss of weight. There was no cough, hemoptysis, night sweats, or contact history of tuberculosis (TB). He denied any arthralgia, rashes, skin eruptions, or alopecia. His bladder and bowel habits were normal. There was no significant travel history or high-risk sexual behavior and he did not have any significant past medical history or significant family history of illnesses. He was initially admitted to the local hospital with fever and lymphadenopathy and he underwent a right-sided cervical lymph node biopsy even though the biopsy report was received later. On day 20 of the fever, he was started on empirical anti-TB treatment, which was stopped after 5 days due to several episodes of loose watery stools. He was admitted to our institution on day 40 of the illness, by which time he had bilateral cervical lumps. On examination he had bilateral firm discrete tender cervical lymphadenopathy and a firm hepatomegaly and mild splenomegaly. The rest of the examination including funduscopy was normal.

The initial laboratory findings are summarized in Table 1.

**Table 1:** The initial biochemical investigations

Investigation	Result	Normal range
Hemoglobin (g/dL)	12.8	11–13
White cell count (10^9^/L)	5.34	4–11
Neutrophil percentage	82%	
Platelet count (10^9^/L)	186	150–450
CRP (mg/L)	27	0–6
ESR (mm/first hour)	64	
Serum creatinine (μmol/L)	91	60–120
Serum sodium (mmol/L)	135	135–148
Serum potassium (mmol/L)	4.0	3.5–5.1
AST (U/L)	217	< 40
ALT (U/L)	154	< 40
ALP (U/L)	133	30–120
Serum total proteins(g/L)	61	61–80
Serum albumin(g/L)	27	36–50
Serum globulin(g/L)	34	22–40

*ALP* alkaline phosphatase, *ALT* alanine aminotransferase, *AST* aspartate aminotransferase, *CRP* C-reactive protein, *ESR* erythrocyte sedimentation rate

Urine analysis was normal and repeated blood cultures, urine cultures, and sputum cultures remained sterile. A Mantoux test was negative. A sputum stain for acid-fast bacilli, TB culture, and GeneXpert TB test were negative. The serology for Epstein–Barr virus (EBV), cytomegalovirus (CMV), *Toxoplasma*, and human immunodeficiency virus (HIV) were negative. Tests for hepatitis B surface antigen and hepatitis C antibody were negative as were antibody tests for *Rickettsiae* and *Brucella*.

**Fig. 1:**
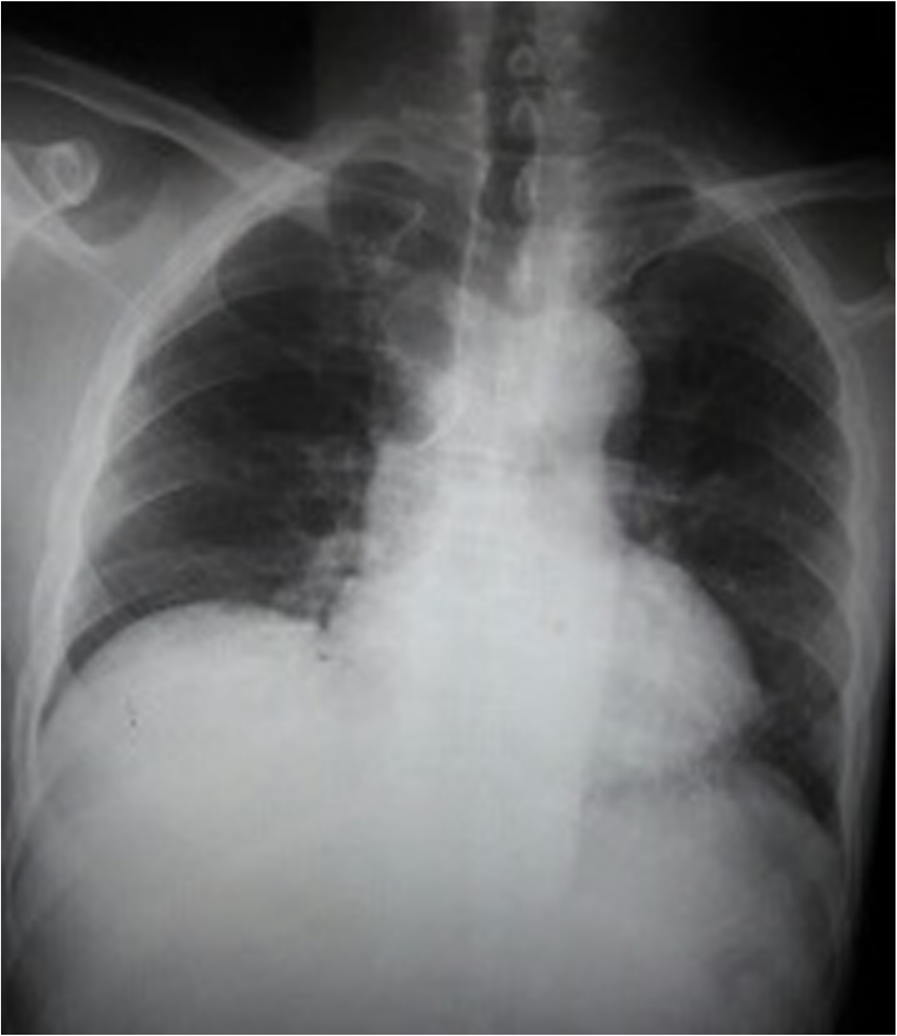
Chest X-ray of the patient, posteroanterior view

A transthoracic echocardiogram and transesophageal echocardiogram did not reveal any vegetation. He had a normal chest X-ray (Fig. 1) and high resolution computed tomography (CT) scan of his chest. An ultrasound scan (USS) of his abdomen revealed a fatty liver with hepatomegaly and a prominent spleen. There was no lymphadenopathy.

A blood picture showed normochromic normocytic red cells with a minor population of hypochromic microcytic red cells and mild leukopenia and thrombocytopenia. Lactate dehydrogenase was 608 U/L. The initial antinuclear antibody (ANA) and anti-double-stranded deoxyribonucleic acid (anti-dsDNA) tests were negative and C3 and C4 levels were normal.

**Fig. 2:**
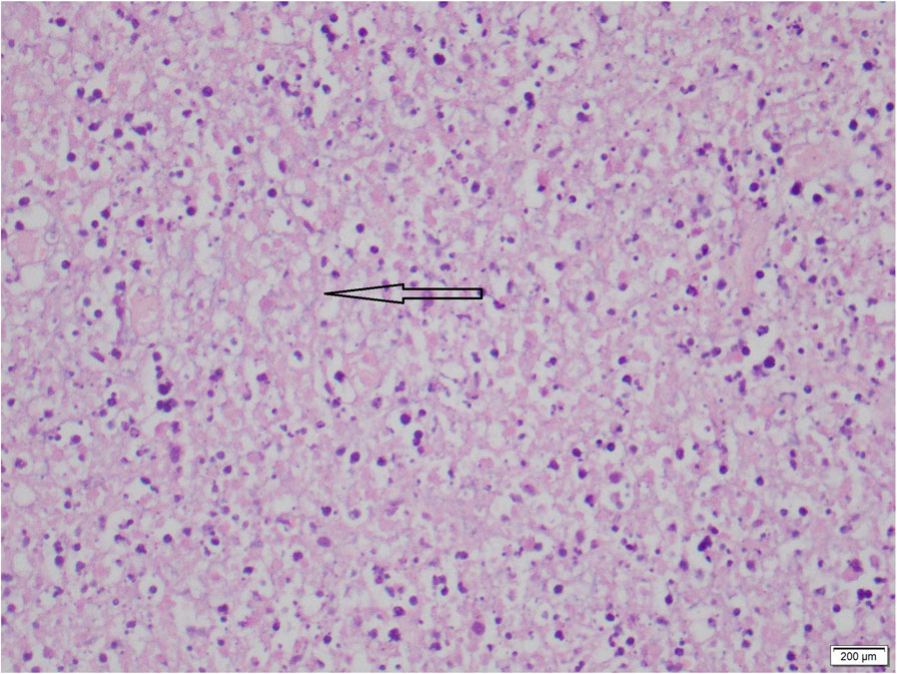
Light microscopy of the lymph node biopsy, hematoxylin and eosin × 400. Necrosis, nuclear debris and plasma cells are present. The arrow is pointing to a plasma cell

**Fig. 3:**
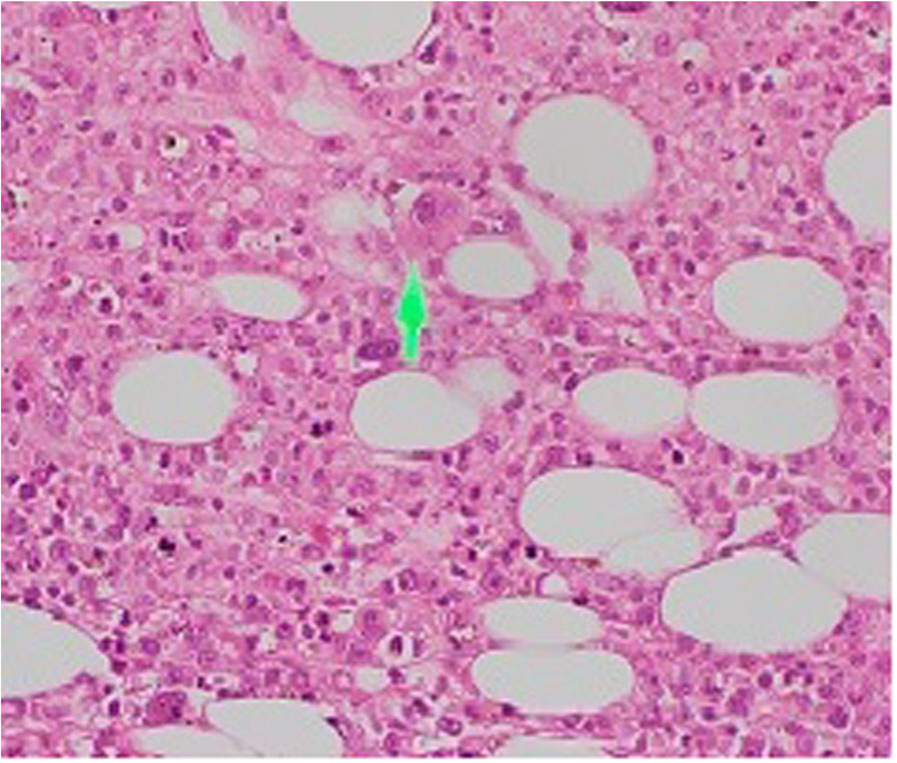
Light microscopy of the lymph node biopsy, hematoxylin and eosin × 400. The arrow is pointing to the foamy histiocytes

**Fig. 4:**
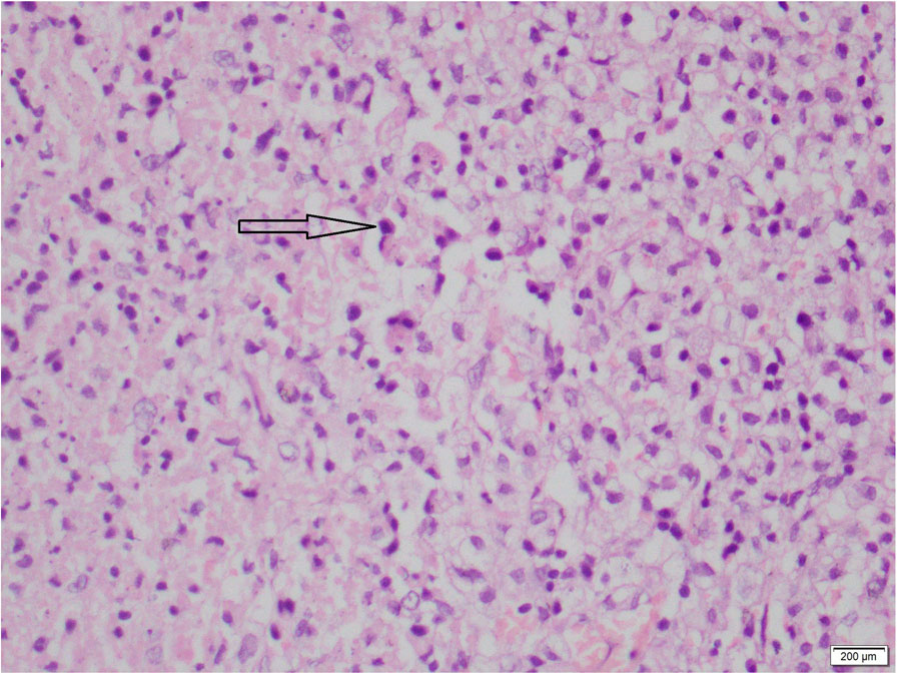
Bone marrow biopsy of the patient showing hemophagocytosis, hematoxylin and eosin × 100. The arrow is pointing to a haemophagocyte

The excision biopsy of the cervical lymph node that was done at the local hospital was available at this point and revealed a partially effaced, normal nodal architecture with scattered lymphoid follicles with germinal centers. There were foci comprising necrosis, nuclear debris (Fig. 2), epithelioid histiocytes (Fig. 3), and lymphocytes; these features were compatible with necrotizing lymphadenitis. Immunohistochemistry showed CD20 to be positive in lymphoid follicles, CD3 to be positive in lymphoid cells in the parafollicular region, and myeloperoxidase (MPO) to be positive in histiocyte-like cells. The presence of MPO positivity, necrotic nuclear debris, and absent neutrophil infiltrate strongly favored KFD. Based on the blood picture and lymph node biopsy result, a bone marrow biopsy (Fig. 4) was performed to rule out lymphoproliferative or hematological malignancies. It revealed cellular marrow with mild granulocytic hyperplasia. There was no evidence of hematological malignancies.

His initial treatment with indomethacin and prednisolone 40 mg daily dose led to fever resolution. However, after 8 days, he developed high fever spikes again. A further laboratory evaluation showed elevated serum ferritin, hypertriglyceridemia, and pancytopenia and there was mild hepatosplenomegaly in a repeat USS of his abdomen. The laboratory findings at this point are shown in Table 2.

**Table 2:** The laboratory findings at the time of diagnosis of hemophagocytic lymphohistiocytosis

Investigation	Result	Normal range
Hemoglobin (g/dL)	6.9	11–13
White cell count(10^9^/L)	1.22	4–11
Neutrophil percentage	43.6%	
Platelet count(10^9^/L)	92	150–450
Serum triglyceride (mg/dl)	288	< 150
Serum ferritin (ng/ml)	4027.4	25–200

A repeat bone marrow biopsy was performed to look for evidence of HLH and it revealed hemophagocytosis. The diagnosis of HLH was made at this point according to the HLH 2004 clinical trial diagnostic guidelines [4]. HLH was presumed secondary to KFD.

He was treated with intravenously administered methylprednisolone pulse therapy 1 g daily for 3 days followed by orally administered prednisolone 60 mg/day. He significantly improved with immunosuppression with steroids. Six weeks from the diagnosis of KFD, he complained of blurring of vision. An ophthalmological evaluation revealed bilateral cotton wool spots, retinal bleeds, and choroidal ischemic changes, which were suggestive of SLE. During this period he also developed acute kidney injury, which required hemodialysis. Antiphospholipid screening, which was done to assess his elevated activated partial thromboplastin time (APTT) and thrombocytopenia to rule out catastrophic antiphospholipid syndrome, was negative. His urine protein creatinine ratio revealed subnephrotic range proteinuria. A repeat ANA titer was positive with a titer of 1:160 in the speckled pattern and the C3/C4 levels were low. A renal biopsy was inconclusive, and he declined a repeat renal biopsy. He also developed a maculopapular rash involving his face, ear lobes, and upper limbs. A skin biopsy showed leukocytoclastic vasculitis. The diagnosis of SLE was made according to the Systemic Lupus Erythematosus International Collaborating Clinics (SLICC) diagnostic criteria. He was started on hydroxychloroquine in addition to orally administered prednisolone. The orally administered prednisolone dose was gradually tapered to a minimum and he showed remarkable improvement in terms of settling of the fever, reduction in the inflammatory marker erythrocyte sedimentation rate (ESR) of 15 mm/first hour), and improvement of pancytopenia.

For timeline of our patient’s clinical course see Table 3.

**Table 3:**
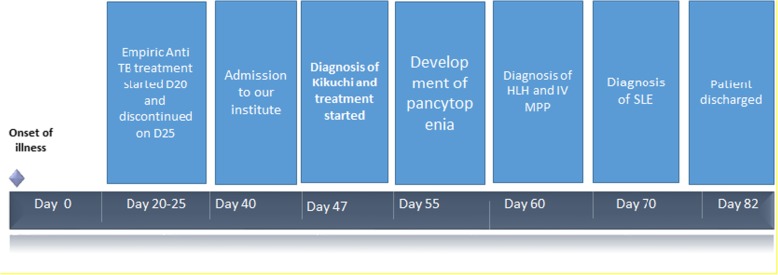
Patient’s clinical course

*D* day, *HLH* hemophagocytic lymphohistiocytosis, *IV* intravenously administered, *MPP* methylprednisolone, *SLE* systemic lupus erythematosus, *TB* tuberculosis

## Discussion

KFD is an uncommon benign condition of necrotizing histiocytic lymphadenitis commonly seen in East Asian and Japanese populations, and it was first described by Kikuchi and Fujimoto in 1972 [2]. Males are affected rarely with a male to female ratio of 1:4 [5]. KFD can be associated with other autoimmune disorders especially SLE [2]. A possible viral etiology was proposed particularly due to its self-limiting nature and nonspecific symptoms. Due to its association with SLE, it is important to look for SLE in patients with KFD as the outcomes may differ when it is associated with SLE. It is sometimes recommended to screen for ANA at the diagnosis of KFD and during follow-up, especially in patients with cutaneous lesions, for the early detection of an autoimmune disease [6].

On day 40 from illness onset, our patient was admitted to our institute with fever of unknown origin and with associated lymphadenopathy for further evaluation and treatment. He had an extensive workup as an out-patient to rule out infectious etiologies. Fever and tender cervical lymphadenopathy are the classic presentations of KFD. However, there are many other possible differential diagnoses such as TB, cat scratch disease, leukemia, and lymphoma; they were excluded by history, examination, and investigations including a bone marrow biopsy. An acid-fast bacilli stain of the lymph node was negative as were the CD30 and anaplastic lymphoma kinase (ALK) stains. His initial ANA done as an out-patient was negative. After the exclusion of other possible differential diagnoses for a similar presentation, the diagnosis of KFD was made based on the lymph node biopsy, histology, and immunohistochemistry. KFD is characterized by focal necrosis in the cortical and paracortical areas associated with marked karyorrhexis and proliferation of the distinctive crescentic histiocytes and plasmacytoid monocytes. The absence of granulocytes, plasma cells, and hematoxylin bodies within the lesions may help to differentiate KFD from lupus lymphadenitis [7]. The lymph node biopsy of our patient revealed a normal nodal architecture with focal necrosis and immunohistochemistry features compatible with KFD.

Our patient developed a fever again despite being on prednisolone 40 mg and indomethacin after the initial response to treatment. He also developed pancytopenia with hepatosplenomegaly on day 55. By that time, all the potential infective causes that could precipitate syndromic manifestation were excluded and other rare possibilities such as HLH and macrophage activation syndrome were suspected. Further laboratory testing revealed hypertriglyceridemia and elevated ferritin. A repeat bone marrow biopsy was performed and it showed evidence of hemophagocytosis. Our patient fulfilled the clinical, laboratory, and histopathology (bone marrow) criteria for HLH (Table 4; our patient’s positive findings are highlighted in yellow) and he was started on high-dose steroids.

**Table 4:**
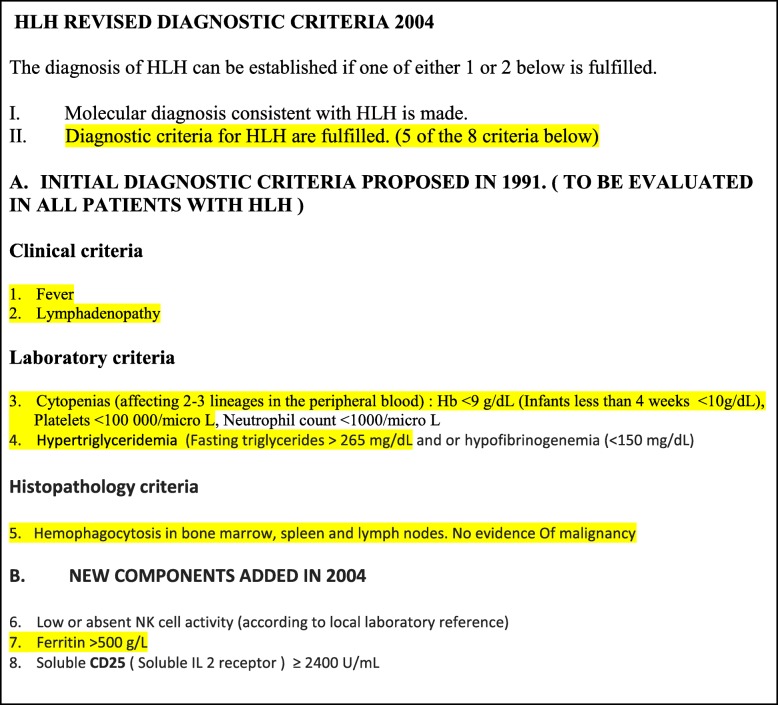
Hemophagocytic lymphohistiocytosis revised diagnostic criteria 2004

*Hb* hemoglobin, *HLH* hemophagocytic lymphohistiocytosis, *IL* interleukin, *NK* natural killer. Our patient’s positive findings are highlighted in *yellow*

HLH is a life-threatening disorder characterized by fever, hepatosplenomegaly, cytopenia, and increased proliferation and activation of benign macrophages with hemophagocytosis throughout the reticuloendothelial system [8]. There are few cases reported with concurrent HLH and KFD and very few reports in which KFD was associated with HLH and SLE simultaneously [7]. There were no case reports on SLE after the onset KFD in a male patient especially with associated HLH to the best of our knowledge. The recognition of this association is of significance for the management of affected patients as they require early intensive immunosuppressive therapy for a favorable outcome [7].

While on high-dose steroids, our patient developed acute kidney injury with proteinuria and leukocytoclastic vasculitis, in addition to the persistent cytopenia and fever. On day 70 SLE was suspected and ANA and complements were tested. SLE was diagnosed on day 70 based on the SLICC criteria [9] for diagnosis of SLE (Table 5). Our patient fulfilled the clinical criteria (thrombocytopenia, leukopenia, and renal failure with proteinuria) and immunological criteria (positive ANA and low complements) with a total of 5 out of 11 criteria.

**Table 5:**
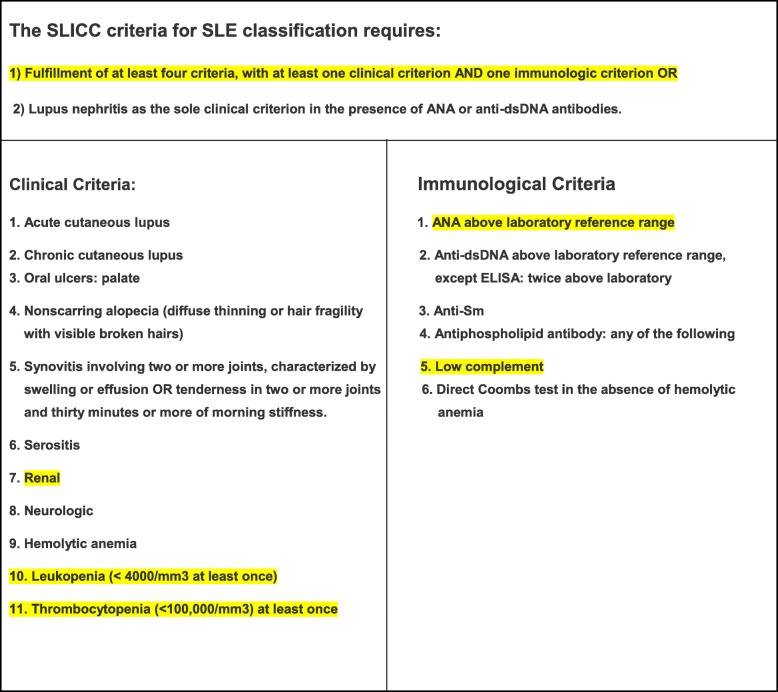
Systemic Lupus Erythematosus International Collaborating Clinics criteria for the diagnosis of systemic lupus erythematosus

*ANA* antinuclear antibody, *dsDNA* double-stranded DNA, *ELISA* enzyme-linked immunosorbent assay, *SLE* systemic lupus erythematosus, *SLICC* Systemic Lupus Erythematosus International Collaborating Clinics Our patient’s positive findings are highlighted in *yellow*

Several reports have emphasized the importance of the association between KFD and SLE as KFD can precede or coexist with SLE. Because of the clinical and pathological correlation between KFD and SLE, some authors have postulated that KFD may be a clinical feature or an incomplete phase of lupus lymphadenitis. However, there are several case reports of KFD without SLE, which may favor them to be two independent entities that commonly coexist, as happens with most autoimmune diseases in susceptible individuals [2].

One could argue how our patient developed SLE while on immunosuppressive therapy and the answer is not entirely clear. However we could postulate a few things:

- ➢ The patient could have developed SLE even prior to high-dose steroid therapy. We did not test ANA when he developed pancytopenia as we were pursuing an alternative diagnosis of HLH.
- ➢ Initial moderate prednisolone therapy and subsequent high-dose therapy could have masked some of the other manifestations of SLE such as classical lupus nephritis and arthritis. Although our patient’s clinical picture was suspicious for glomerulonephritis, a biopsy was not conclusive. The biopsy could have been affected by steroid therapy as well.
- ➢ Anti-TB drugs such as isoniazid are known to precipitate drug-induced lupus; however, to date we have not found any literature to support it precipitating SLE.

## Conclusion

Although KFD is uncommon in male patients, it should be considered in patients with lymphadenopathy and fever. The disease can be complicated with HLH and patients need continuous monitoring for the possible development of SLE later in the course.
